# Explanations on school task procrastination reported by medical students: A qualitative study at a public university in South-eastern Brazil

**DOI:** 10.1192/j.eurpsy.2022.1780

**Published:** 2022-09-01

**Authors:** E. Turato, L. Gonzalez

**Affiliations:** State University of Campinas, Laboratory Of Clinical-qualitative Research, Campinas, Brazil

**Keywords:** Qualitative research, procrastination, mental health care, medical students

## Abstract

**Introduction:**

How do medical students, who have self-criticism of being procrastinators of their study obligations, deal psychologically with daily tasks? The experience of procrastination by those who are considered high-performance students involves resources of mental health to cope with guilt, exhaustion, or even self-sabotage. According to MeSH used by PubMed, procrastination is ‘the deferment of actions or tasks to a later time, or to infinity’.

**Objectives:**

To explore the psychological meanings that medical students attribute to procrastination phenomena to better understand how they handle the usual curriculum overload.

**Methods:**

Clinical-qualitative design. Sample of 13 participants closed by information saturation with 2nd, 3rd, 4th-year students. Semi-directed interviews with open-ended questions in-depth. Clinical-qualitative content analysis, free-floating readings with psychodynamic concepts. Results were validated by peers at the Laboratory of Clinical-Qualitative Research.

**Results:**

Emergent categories: 1) between procrastination and despair: the process of stress in procrastination; 2) a proving mechanism: procrastination as an emotional defense; 3) a very delicate rumination: between procrastination and mere delay, an emotional dilemma; 4) this conflict is painful: the confrontation between the desire to comply with tasks with excellence and the enjoyment of life.

**Conclusions:**

Procrastination is reported by students as a source of great tension generated by opposing forces and desires, in which exhaustion is eventually reached. There are emotional contradictions related to guilt for leaving tasks to the last moment and the need to live other things besides doing academic tasks. Procrastination is a message-metaphor. It is important that institutions listen to students to understand what procrastination is saying about them.

**Disclosure:**

No significant relationships.

